# The Role of Polyether Ether Ketone (Peek) in Dentistry – A Review

**DOI:** 10.25122/jml-2019-0003

**Published:** 2019

**Authors:** Lakshmana Bathala, Vaishnavi Majeti, Narendra Rachuri, Nibha Singh, Sirisha Gedela

**Affiliations:** 1. Department of Prosthodontics, Lenora Institute of Dental Sciences, Rajahmundry, India; 2. Department of Oral & Maxillofacial Surgery, Lenora Institute of Dental Sciences, Rajahmundry, India

**Keywords:** Polyether Ether Ketone (PEEK), PEEK Implants, Modified PEEK polymer, Carbon Fiber Reinforced -Poly Ether Ether Ketone (CFR-PEEK), Telescopic Crowns, Obturator, PEEK - Polyether Ether Ketone, CFR - PEEK- Carbon Fiber Reinforced - Polyether Ether Ketone, RPD-Removable Partial Denture, FPD - Fixed Partial Denture, MFP - Maxillofacial Prosthesis

## Abstract

This study is aimed to review the applications of Polyether Ether Ketone (PEEK) in dentistry. The increased demand for aesthetics, legislation in some developed countries, few drawbacks with existing materials and clinicians shifting their paradigms towards metal free restorations led space for the metal-free restorations in today’s dental practice. An electronic literature search was conducted through Medline via PubMed, Wiley Online library, EBSCOhost, Science Direct, as well as the Google Scholar between January 2010 and March 2018 using the keywords: PEEK, modified PEEK, PEEK and Dental, advantages of PEEK, applications of PEEK in dentistry and PEEK Implants. A total of 103 articles were found in the literature search and out of these, 18 were not related to our study and hence were excluded. Finally, 85 articles were found to be relevant. PEEK has been explained for a number of applications in dental practice. The literature showed that the PEEK material has superior mechanical properties with different uses in various specialties of dentistry.

## Introduction

The long term success of dental implant depends mainly on minimizing the amount of marginal bone loss on functional loading. Titanium and its alloys and Zirconium are predominant in the field of implant materials in today’s dental practice. Studies have already proven that these materials are biocompatible, but even these have some short comes, one of them being the elastic modulus. The elastic modulus of titanium and zirconia are 110 and 210 GPa respectively which is 5-14 times greater than that of compact bone having 15 GPa [1, 2]. Due to the gradient difference in the elastic modulus of a titanium implant to its surrounding bone, it may cause stress in the implant-bone interface during load transfer resulting in peri-implant bone loss [2]. This phenomenon is referred to as stress shielding, and it may be one of the important causes of long term failure of dental implants. Titanium implants are also known to cause image distortions in MRI scans [3]. Few studies also claimed that titanium is prone to hypersensitivity reactions [4]. Titanium can cause aesthetic problems due to its lack of light transmission [5]. This can provoke a dark shimmer of the peri-implant soft tissue in cases of thin biotype mucosa and mucosa recession around the implant [6]. The existing materials despite having superior qualities have certain drawbacks like attrition of the natural teeth and bulkiness which may lead to a compromise in the retention of the prosthesis as well as patient satisfaction. The dental profession always thrives for better materials which can fulfill the pitfalls of the existing materials. PEEK is the latest inventory of dentistry and is claimed to have better properties in parallel with existing materials. Therefore, keeping all these in mind, in this review, we aimed to focus exclusively on the mechanical properties, advantages, modifications of the PEEK material and different uses of PEEK in various specialties of dentistry.

## Materials and Methods

An electronic literature search was conducted through Medline via PubMed, Wiley Online library, EBSCOhost, Science Direct, as well as Google Scholar between January 2010 and November 2018 using the keywords PEEK, modified PEEK, PEEK and Dental, advantages of PEEK, applications of PEEK in dentistry and PEEK implants. The articles in the English language were the only ones considered. A total of 103 articles were found but 18 were not related to our study, and they were excluded. Abstracts, short communications and company literature were excluded as well. Finally, 85 articles were found to be relevant for our search.

## Results

There were a total of 85 articles found in our research after exclusion criteria. The data showed that the distribution of articles on the properties of PEEK was 17 (20%), implant-related articles were 26 (30.6%) whereas the maximum number of articles related to different prosthodontic applications were 33 (38.9%) and 9 (10.6%) articles found on the usage of PEEK in other specialties of dentistry which includes orthodontics, oral and maxillofacial surgery, and endodontics. The 33 articles which belong to prosthodontics,18 of them are related to different Crown systems (54.5%), 6 are removable partial dentures (18%), 5 are fixed partial dentures (15%), and 4 were in relation to maxillofacial prosthodontics (12%) (Table 1).

**Table 1:** Distribution of PEEK articles in relation to Dentistry

S.no	Categorization of Distribution of PEEK articles	Number of articles (N=85)
1.	Properties of PEEK	17 (20%)
2.	Implants	26(30.6%)
3.	Prosthodontic Applications	33 (38.9%)
	A. Different types of Crowns	18 (54.5%)
	B. Removable Partial Dentures (RPD)	6 (18%)
	C. Fixed Partial Dentures (FPD)	5 (15%)
	D. Maxillofacial Prosthesis (MFP)	4 (12%)
4.	Applications in other Specialities of dentistry (Orthodontics, Oral and Maxillofacial Surgery and Endodontics)	09 (10.6%)

## Discussion

There are 20% of articles related to the properties of PEEK, which revealed the superior mechanical properties over the existing metals used in dentistry. PEEK is a high-temperature thermoplastic, semi-crystalline material with high melting temperature. Documented evidence suggests the physical properties such as the elastic modulus of PEEK is 3.6 GPa, and by incorporating carbon fibers, the elastic modulus can be improved to 18 GPa which is close to that of cortical bone, i.e., 15GPa [4,7,8]. Being radiolucent, PEEK has reduced magnetic resonance imaging artifacts and is very rigid with a flexural strength of 140-170 MPa [4, 8–14]. Another great advantage of PEEK is that it does not attrite the opposing natural teeth. Its biocompatibility and bio-stability are supported by the US FDA Drug & Device Master files [12]. Bio HPP, the modified form of PEEK is more advantageous for being anti-allergic in nature, non-metallic in taste, excellent polishing properties, low plaque affinity, and good wear resistance. It can also be used as an alternative RPD framework material when mixed with regular acrylic denture teeth and denture base material. PEEK with its low specific weight can be used to construct very lightweight prosthesis which will provide high patient satisfaction and comfort. Its hygienic design as well simplifies, and it helps to maintain oral hygiene [13]. The articles on properties have revealed the superiority of PEEK material over the other materials and this made PEEK to be considered as a substitute for the other materials that are currently used in dentistry.

PEEK modified with nanometer zirconia has shown the lowest wear property and friction resistance compared with pure PEEK [15,16]. In recent years, PEEK has also been modified at the nano-level in order to improve its bioactivity and osseoconductive properties. The nanoparticles like TiO2, HAF and HAp, can be combined with PEEK to develop bioactive nanocomposites [17]. Kartzer et al. mentioned that there is no evidence of either mutagenicity or cytotoxicity with PEEK on the human organs and this shows that PEEK has good biocompatible properties [18].

PEEK was described as an alternative material for implants by 30.6% of the articles. According to Beuer et al., the fracture resistance of PEEK is higher than zirconia and ceramics, and PEEK can be modified easily by incorporation of other materials [19]. For example, incorporation of carbon fibers to increase the elastic modulus up to 18 GPa and thus the elasticity of this material might reduce the distal torque and the stress on the abutment teeth. When reinforced with fiber, PEEK can reduce stress shielding when compared with traditional metallic implants [9]. The modulus of carbon-reinforced PEEK is also comparable to those of cortical bone and dentin [11,20,21]. Bio HPP can be considered as a good alternative material for abutments with decreased periodontal support when replacing distal extension situations. However, there is still not enough information available about complications or biofilm formation on a PEEK surface. The PEEK polymer can exhibit lesser stress shielding when compared to titanium which is presently dominating the implant dentistry. According to Sano H et al. [22] and Sandler J et al. [23], the tensile properties of PEEK are also analogous to that of bone, enamel, and dentin. Thus, this material can exhibit less stress shielding effect compared to titanium, and it can be considered as a good substitute material. The surface modification of PEEK with Tantalum ions improves the modulus of elasticity closer to cortical bone and improves the osseointegration [24]. Many researchers claimed that the long term success of dental implants depends mainly on minimizing the amount of marginal bone loss after several years of functional loading. This is mainly depending on the implanted biomaterial which has to meet the mechanical property of the host bone tissue [25–27]. Sagomonyants et al. [28] claimed in their finite element analysis (FEA) that CFR-PEEK implants could induce lesser stress shielding than titanium, whereas another FEA study results by Sarot et al. [2] contradicts the above study. Hence, some more clinical trials are required to prove bone shielding effect of PEEK.

PEEK/Nano-FHA composite has shown better biocompatibility and antibacterial activity with better osseointegration[15]. The results of a pilot study by Stubinger and colleagues [29] on osseointegration capability of PEEK and CFR-PEEK implants coated with titanium and hydroxyapatite plasma sprayed layers, revealed that both materials with surface coatings had increased osseointegration. More favorable results were seen when double coated using titanium bond layer and hydroxyapatite top layer after 2 and 12 weeks. Becker et al. described a novel method regarding the use of prefabricated PEEK abutment screwed into the internal connection of the implant to get the proper emergence profile [30]. This avoids surgical procedure before impression making which in turn reduces chairside time. The multi-layered nanoporous surface modification with TiO2 on CFR-PEEK improves the elastic recovery, stability and enhances as well the antibacterial activity [31]. According to Barkarmo et al., there has been much focus on the nanoscale coating of PEEK with bioactive apatite [32]. As mentioned by Almasi et al., the deposition of HA via plasma spraying method is the best one [33].

There are 38.9% articles that mentioned about the role of PEEK and its different uses in prosthodontics and 10.6% on usage in various specialties of dentistry which gives that the modified PEEK has been promising for the future trends. The fatigue strength for BioHPP is very high (1200N) and seems to be suitable as superstructures for implants, implant-supported bars and clamp for removable prostheses, provisional abutments for fixed dental prosthesis [34]. PEEK reinforced with other inorganic fillers can be potentially used as crown and bridge material [35]. However, unmodified PEEK is inherently hydrophobic, with a contact angle of 80–900 and is bioinert [36]. Hence the unmodified PEEK requires the addition of fillers to reduce contact angle so that it can acquire hydrophilic characters.

The bioactive nanocomposites can be used as indirect intracoronal or extracoronal restorations. According to Wang et al., these restorations can have an additional advantage of being antibacterial [25]. By using computer-aided design and computer-aided manufacturing systems, PEEK dentures can be constructed [9]. The denture clasps made of PEEK have a lower retentive force compared to Cobalt–Chromium (Co–Cr) clasps [37]. Costa-Palau et al. [38] mentioned in their clinical report regarding the fabrication of maxillary obturator for a patient with oro-nasal defect using PEEK as an alternative to conventional materials and methods. They claimed that the PEEK obturator is weightless, biocompatible, with good retention and ease of polishing. CAD-CAM prepared for 3-unit FPD has more fracture resistance than the conventional methods [39]. The chairside methods have less surface roughness values than laboratory methods [40].

According to Stawarczyk et al. [39] and Skirbutis et al. [41] due to the greyish brown color of PEEK, itis not suitable for monolithic aesthetic restorations on anterior teeth. Hence more aesthetic material like composite should be used as a coating to get an aesthetic result. Many surface conditioning methods of PEEK to improve bonding with resin composite crowns has been suggested [42,43]. Being a soft and ductile material, PEEK can yield nicely and adapt well. The adaption process resulted in a good marginal fit [44]. For retention load, PEEK will be a suitable material as a telescopic crown over Zirconia crowns [45]. As an intraradicular post-core material, PEEK shows high fracture resistance compared with metal and fiberglass post-system. Root fracture at the intraradicular surface is the least possible as per the FEA study on the biomechanical property of PEEK conducted by Lee and his co-workers [46]. The PEEK polymer has also joined in line with other gold standards for core materials [47].

CAD/CAM provisional crowns made up of with PEEK material have shown better marginal fit and good fracture strength [48]. The framework for FPD with PEEK provided with very high patient comfort and acceptability [49]. To increase the flexibility of NiTi wires, Boccaccini and his fellow researchers [50] suggested that application of PEEK and PEEK Bioglass particles on a NiTi wire will give better results and also these materials show good adhesion to metallic substrate as well. The conditioned PEEK crowns show the highest values of retention when cemented to dentin abutments after surface modifications [43].

## Conclusion

The PEEK material can be considered to be promising in the future as an alternative to metals like Titanium and Zirconium, due to its high-quality mechanical properties. The modified PEEK materials have shown better properties than the unmodified form of PEEK. Due to its elastic modulus, strength, rigidity, and lightweight, the applications of PEEK is not just limited to implant materials only but also in different clinical situations in dental practice. Though the PEEK is already being used as forerunner material in the spine, orthopedic and sports medicine, the usage of PEEK polymer material in dental practice it has yet to gain momentum. This may be because of the very few long term clinical studies available on the usage of PEEK in the clinical dental practice. Hence more research is needed on PEEK polymer as an alternative material for existing metals which are have been used for a long time.

## Conflict of Interest

The authors confirm that there are no conflicts of interest.
